# The risk and predictors for severe radiation pneumonitis in lung cancer patients treated with thoracic reirradiation

**DOI:** 10.1186/s13014-018-1016-z

**Published:** 2018-04-16

**Authors:** Chengbo Ren, Tianlong Ji, Tingting Liu, Jun Dang, Guang Li

**Affiliations:** Department of Radiation Oncology, The First Hospital of China Medical University, 155 Nanjing Road, Heping District, Shenyang, 110001 China

**Keywords:** Lung cancer, Reirradiation, Radiation pneumonitis, Predictors

## Abstract

**Background:**

Thoracic reirradiation (re-RT) is increasingly administered. However, radiation pneumonitis (RP) remains to be the most common side effect from retreatment. This study aimed to determine the risk and predictors for severe RP in patients receiving thoracic re-RT.

**Methods:**

Sixty seven patients with lung cancer received thoracic re-RT for recurrent or metastatic disease. Three-dimensional conformal radiotherapy (3D-CRT)/intensity modulated radiotherapy (IMRT) was used for 60 patients, and stereotactic body radiation therapy (SBRT) was used in 7 patients. Deformable image registration (DIR) was performed to create a composite plan. Severe (grade ≥ 3) RP was graded according to Common Terminology Criteria for Adverse Events version 4.0.

**Results:**

Eighteen patients (26.9%) developed grade ≥ 3 RP (17 of grade 3, and 1 of grade 4). In univariate analyses, V5 and mean lung dose (MLD) of initial RT or re-RT plans, V5 and V20 of composite plans, and the overlap between V5 of initial RT and V5 of re-RT plans/V5 of re-RT plans (overlap-V5/re-V5) were significantly associated with grade ≥ 3 RP (*P* < 0.05 for each comparison). Multivariate analysis revealed that MLD of the initial RT plans (HR = 14.515, 95%CI:1.778–118.494, *P* = 0.013), V5 of the composite plans (HR = 7.398, 95%CI:1.319–41.495, *P* = 0.023), and overlap-V5/re-V5 (*P* = 0.041) were independent predictors for grade ≥ 3 RP. Out-of-field failures with medium overlap-V5/re-V5 of 0.4–0.8 was associated with higher risk of grade ≥ 3 RP compared with in-field failures (18.3% vs. 50%, *P* = 0.014).

**Conclusions:**

The risk of grade ≥ 3 RP could be predicted not only by dose-volume variables from re-RT plan, but also by some from initial-RT and composite plans. Out-of-field failures was associated with higher risk of severe RP compared with in-field failures in some cases.

## Background

Lung cancer is the most prevalent malignancies and the leading cause of cancer death worldwide. Radiotherapy has an important role in the radical treatment of early and advanced lung cancer. However, the rates of recurrent disease after RT still remain high [1, 2]. For recurrent pulmonary tumors after previous thoracic RT, salvage surgery is typically avoided. Second line chemotherapy for such patients has been assessed in several studies, unfortunately the objective response rates and survival are quite poor [3–5]. With technologic advances, there is growing interest in the use of reirradiation (re-RT) for such diseases. Published studies have demonstrated the effectiveness of re-RT for recurrent, metastatic pulmonary tumors, or a new primary lung tumor after previous thoracic RT [6–14]. However, radiation pneumonitis (RP) remains to be the most common side effect from retreatment, up to 40% in some cases [6, 12, 14].

Data for dose-volume predictors of severe RP in patients undergoing thoracic re-RT are scarce. V20 ≥ 30% of the composite plans was reported to be associated with severe RP in one small retrospective study [15]. However, deformable image registration (DIR) [16, 17] was not used to generate the composite plans in that study, leading to less accuracy for assessing the composite doses to lung. Besides, re-RT may cause some overlap with the previously irradiated lung volumes. Whether the overlap is related to the risk of RP remains unclear. Moreover, the effect of re-RT characteristics (such as re-RT technique, re-RT location, and Interval between initial and re-RT) on the incidence of RP has not been investigated sufficiently.

In light of these critical issues, we retrospectively investigated the risk and predictors of severe RP in patients undergoing thoracic re-RT.

## Methods

### Patients

Between January 2010 and January 2017, 70 patients with lung cancer who had previously received thoracic RT were retreated with RT to the thorax due to recurrent, metastatic, or new primary lung tumors. Three patients receiving initial RT at outside institutions were excluded because they could not obtain the pretreatment imagings and treatment plans. Thus, 67 patients were included in this study. Diagnosis of them mainly used bronchoscopy, CT-guided transthoracic needle aspiration biopsy, or based on FDG-PET/CT scans.

### Radiation treatment

Re-RT was delivered using conventional three-dimensional conformal radiotherapy (3D-CRT)/intensity modulated radiotherapy (IMRT) or stereotactic body radiation therapy (SBRT). 6-MV x-rays were used. The prescribed dose was 40–60 Gy in 2.0 Gy daily fractions for 5 days a week in patients treated with conventional RT and 40–50 Gy in 8–10 Gy daily fractions for 3 days a week in those receiving SBRT. For conventional RT treated patients, gross tumor volume (GTV) was defined as the total volume of the tumor visualized on planning CT image, and was expanded to create an internal target volume (ITV), and a planning target volume (PTV). For patients receiving SBRT, GTV was delineated on a maximal intensity projection of 4-dimensional computed tomography (4D-CT) and modified according to its movement to create the internal gross tumor volume (iGTV), and an expansion of 3–5 mm was added to create a PTV. One hundred percent of the prescription dose covered 95% of the PTV.

Initial and Re-RT planning CT scans and their respective planned doses were imported into MIM Maestro Software (version 6.5.9). DIR was performed to create a composite plan [16, 17]. The following dose-volume histogram (DVH) data were extracted from the initial RT, re-RT, and composite plans, respectively: mean lung dose (MLD), lung V5, V20, and V50. The overlap between V5 of initial RT and V5 of re-RT plans was measured and the ratio of the overlap to V5 of re-RT plans (overlap-V5/re-V5) was calculated. For patients treated with SBRT, radiation dose was converted to an equivalent dose in 2 Gy fractions (EQD2, α/β = 3 Gy for lung and α/β = 10 Gy for tumor). Re-RT tumors in which the geometric center of the mass was within the initial 50% prescribed dose line were defined as in-field failures.

### Follow-up and RP evaluation

Patients were evaluated weekly during re-RT, every 2 months for the first year after re-RT, and every 3–6 months thereafter. A diagnosis of RP was made with consensus by at least two radiation oncologists. RP was graded according to the National Cancer Institute Common Terminology Criteria for Adverse Events (CTCAE) version 4.0 [18]. Criteria for grade 3 and 4 were as follows. Grade 3, patient manifested severe symptoms limiting self-care ADL. Oxygen has been indicated; grade 4, patient manifested life-threatening respiratory compromise with urgent intervention indicated (e.g. tracheotomy and intubation).

### Statistical analysis

Grade ≥ 3 RP was counted as event. The SPSS 24.0 statistical software package (Chicago, IL) was used to analyze the data. All continuous variables were dichotomized according to the sample median (except overlap-V5/re-V5) and then analyzed as nominal categorical variables. Patient characteristics and dose-volume variables from 3 sets of plans were assessed for correlations with the risk for grade ≥ 3 RP. Univariate analysis was done with Pearson’s χ^2^ or Fisher’s exact tests. Variables with a *P* < 0.05 on univariate analysis were then entered in a stepwise method in a binary logistic regression analysis to develop a multivariate model of independent factors predicting grade ≥ 3 RP. Linear-by-linear association testing was used to determine a correlation between location of re-RT (in-field/out-of-field) and overlap-V5/re-V5 (0.8–1/0–0.8). *P* < 0.05 was considered statistically significant.

## Results

### Patient, tumor, and treatment characteristics

Patient characteristics are listed in Table 1. Of the 67 patients in this study, 63 (94%) completed reirradiation as planned. FDG-PET/CT was mainly used for the diagnosis of recurrent, metastatic, or new primary lung tumors, and histological confirmation was achieved in 38.8% (26/67) of the patients. The median follow-up time after re-RT was 9 months (range, 4–76 months). Median time to Re-RT was 16 months (range, 2–96 months). Most patients received conventional 3D-CRT/IMRT either in initial RT (*n* = 65, 97.0%) or re-RT (*n* = 60, 89.6%). The median dose were 56 Gy (α/β = 10; range, 30–120 Gy) and 54 Gy (α/β = 3; range, 30–260 Gy) for initial RT, and were 54 Gy (α/β = 10; range, 14–106 Gy) and 54 Gy (α/β = 3; range, 14–240 Gy) for re-RT. Nineteen patients (28.4%) received chemotherapy with re-RT including induction (*n* = 5), or concurrent (*n* = 5), or sequential chemotherapy (*n* = 12). The regimens most often given for patients with previous NSCLC were taxanes and carboplatin or cisplatin, while the regimens given for patients with previous SCLC were various.

**Table 1:** Patient characteristics

Variable	Value or No. of patients(%)
Sex	
Male	48 (71.6%)
Female	19 (28.4%)
Median age (years, range)	59 (34~ 80)
Current smoker	
Yes	43 (64.2%)
No	24 (35.8%)
Tumor type	
Recurrences	54 (80.6%)
Metastases	10 (14.9%)
New primary tumors	3 (4.5%)
Pathologic type	
NSCLC	36 (53.7%)
SCLC	31 (46.3%)
Prior thoracic surgery	
Yes	17 (25.4%)
No	50 (74.6%)
ECOG status before Re-RT	
0–1	61 (91.0%)
2	6 (9.0%)
Chemotherapy	
Yes	19 (28.4%)
No	48 (71.6%)
Initial RT technique	
Conventional	65 (97.0%)
SBRT	2 (3.0%)
Re-RT technique	
Conventional	60 (89.6%)
SBRT	7 (10.4%)
Re-RT location	
Central	47 (70.1%)
Peripheral	20 (29.9%)
Re-RT location	
In field	33 (49.3%)
Outside	34 (50.7%)
Median time to Re-RT(months, range)	16 (2–96)
Median follow-up(months, range)	9(4–76)

*Abbreviations*: *NSCLC* non-small cell lung cancer, *SCLC* small cell lung cancer, *Re-RT* reirradiation, *SBRT* stereotactic ablative radiation therapy

### Risk factors for grade ≥ 3 RP

Grade 0–1 and 2 RP were observed in 39 (58.2%) and 10 (14.9%) patients, resoectively. Grade ≥ 3 RP was observed in 18 patients (26.9%), consisting of 17 patients with grade 3 and 1 patient with grade 4. No grade 5 RP was noted.

In univariate analyses, V5 and MLD of initial RT or re-RT plans, V5 and V20 of composite plans, and overlap-V5/re-V5 were significantly associated with grade ≥ 3 RP (*P* < 0.05 for each comparison) (Tables 2 and 3). All of the variables showing significant associations on univariate analysis were then entered in a multivariate logistic regression analysis with stepwise variable selection (Table 4). In that analysis, MLD of the initial RT (initial-MLD) (HR = 14.515, 95%CI:1.778–118.494, *P* = 0.013), V5 of the composite plans (composite-V5) (HR = 7.398, 95%CI:1.319–41.495, *P* = 0.023), and overlap-V5/re-V5 (*P* = 0.041) were independent predictors for grade ≥ 3 RP.

**Table 2:** Univariate analysis of associations between patient characteristics and grade ≥ 3 RP (*n* = 67)

Variable	Grade 0–2	Grade ≥ 3	*P* value
	(*N* = 49)	(*N* = 18)	
Sex			0.584
Male	36 (73.5%)	12 (66.7%)	
Female	13 (26.5%)	6 (33.3%)	
Age			0.822
≥ 65	15 (30.6%)	5 (27.8%)	
< 65	34 (69.4%)	13 (72.2%)	
Smoking			0.797
Yes	31 (63.3%)	12 (66.7%)	
No	18 (36.7%)	6 (33.3%)	
Pathologic type			0.856
NSCLC	26 (53.1%)	10 (55.6%)	
SCLC	23 (46.9%)	8 (44.4%)	
Prior thoracic surgery			0.364
Yes	11 (16.4%)	6 (9%)	
No	38 (56.7%)	12 (17.9%)	
ECOG PS before Re-RT			0.914
0–1	44 (89.9%)	17 (94.4%)	
2	5 (10.2%)	1 (5.6%)	
Chemotherapy			0.246
Yes	12 (24.5%)	7 (38.9%)	
No	37 (75.5%)	11 (61.1%)	
Median Time to Re-RT			0.114
≥ 15.57	22 (44.9%)	12 (66.7%)	
< 15.57	27 (55.1%)	6 (33.3%)	
Initial technique			1
Conventional	47 (95.9%)	18 (100%)	
SBRT	2 (4.1%)	0 (0%)	
Re-RT technique			0.213
Conventional	42 (85.7%)	18 (100%)	
SBRT	7 (14.3%)	0 (0%)	
Re-RT location			0.822
Central	34 (69.4%)	13 (72.2%)	
Peripheral	15 (30.6%)	5 (27.8%)	
Re-RT location			0.114
In field	27 (55.1%)	6 (33.3%)	
Outside	22 (44.9%)	12 (66.7%)	

*Abbreviations*: *RP* radiation pneumonitis, *NSCLC* non-small cell lung cancer, *SCLC* small cell lung cancer, *SBRT* stereotactic ablative radiation therapy, *Re-RT* reirradiation

**Table 3:** Univariate analysis of associations between dosimetric factors and grade ≥ 3 RP (*n* = 67)

Variable	Grade 0–2 (*n* = 49)	Grade ≥ 3 (*n* = 18)	*P* value
Initial-V5			0.047
≥ 53.15%	22 (44.9%)	13 (72.2%)	
< 53.15%	27 (55.1%)	5 (27.8%)	
Initial-V20			0.152
≥ 18.04%	23 (46.9%)	12 (66.7%)	
< 18.04%	26 (53.1%)	6 (33.3%)	
Initial-V50			0.633
≥ 2.04%	24 (49.0%)	10 (55.6%)	
< 2.04%	25 (51.0%)	8 (44.4%)	
Initial-MLD			0.002
≥ 10.87	20 (40.8%)	15 (83.3%)	
< 10.87	29 (59.2%)	3 (16.7%)	
Re-V5			0.001
≥ 32.7%	19 (38.8%)	15 (83.3%)	
< 32.7%	30 (61.1%)	3 (16.9%)	
Re-V20			0.114
≥ 9.94%	22 (44.9%)	12 (66.7%)	
< 9.94%	27 (55.1%)	6 (33.3%)	
Re-V50			0.532
≥ 1.25%	26 (53.1%)	8 (44.4%)	
< 1.25%	23 (46.9%)	10 (55.6%)	
Re-MLD			0.001
≥ 6.63	19 (38.8%)	15 (83.3%)	
< 6.63	30 (61.2%)	13 (16.7%)	
Composite-V5			< 0.001
≥ 68.13%	18 (36.7%)	16 (88.9%)	
< 68.13%	31 (63.3%)	2 (11.1%)	
Composite-V20			0.033
≥ 28.04%	21 (42.9%)	13 (72.2%)	
< 28.04%	28 (57.1%)	5 (27.8%)	
Composite-V50			0.304
≥ 7.23%	23 (46.9%)	11 (61.1%)	
< 7.23%	26 (53.1%)	7 (38.9%)	
Composite-MLD			0.114
≥ 17.31	22 (44.9%)	12 (66.7%)	
< 17.31	27 (55.1%)	6 (33.3%)	
Overlap-V5/Re-V5			0.035
0–0.4	12 (24.5%)	2 (11.1%)	1.000*
0.4–0.8	10 (20.4%)	10 (55.6%)	0.014*
0.8–1	27 (55.1%)	6 (33.3%)	

*Abbreviations*: *RP* radiation pneumonitis, *Initial* initial radiation plan, *MLD* mean lung dose, *Re* reirradiation plan, *Composite* composite plan, *Overlap-V5/Re-V5* the overlap between V5 of initial RT and V5 of re-RT plans/V5 of re-RT plans, *Vx* percent volume of lung exposed to at least x Gy, * compared with overlap-V5/Re-V5 of 0.8–1

**Table 4:** Multivariate analysis of factors associated with grade ≥ 3 RP

Variable	OR(95CI)	*P* value
Initial-MLD	14.515 (1.778–118.494)	0.013
Composite-V5	7.398 (1.319–41.495)	0.023
OverlapV5/ReV5		0.041
0–0.4	5.564 (0.382–81.023)	0.209
0.4–0.8	11.01 (1.71–70.881)	0.012
0.8–1	1	

*Abbreviations*: *RP* radiation pneumonitis, *Initial* initial radiation plan, *MLD* mean lung dose, *Composite* composite plan, *Overlap-V5/Re-V5* the overlap between V5 of initial RT and V5 of re-RT plans/V5 of re-RT plans, *Vx* percent volume of lung exposed to at least x Gy

There was no significant difference in the risk of grade ≥ 3 RP between patients with overlap-V5/re-V5 of 0.8–1 (18.2%) and patients with overlap-V5/re-V5 of 0–0.8 (33.3%) (*P* = 0.114). But, rates of grade ≥ 3 RP for patients with overlap-V5/re-V5 of 0.8–1 were significantly lower when compared with those with overlap-V5/re-V5 of 0.4–0.8 (50%) (*P* = 0.014), and was similar with those with overlap-V5/re-V5 of 0–0.4 (14.3%) (*P* = 1.000).

Patients with In-field relapses had a relatively lower rates of grade ≥ 3 RP compared with out-of-field failures (18.2% vs. 35.3%), but with no significant statistical difference (*P* = 0.114). In-field relapses (87.9%) were more frequently observed in patients with higher overlap-V5/re-V5 (0.8–1) and out-of-field relapses (87.2%) in patients with lower overlap-V5/re-V5 (0–0.8). Linear-by-linear association testing revealed that there was significant correlation between location of re-RT (in-field/out-of-field) and overlap-V5/re-V5 (0.8–1/0–0.8) (*P* < 0.001).

## Discussion

Published data have suggested that either thoracic re-RT with conventional fractionation [8–10, 19, 20] or with SBRT [5–7, 11–14] is a reasonable treatment option to consider for relapse or progression. However, there is a lack of prospective evidence detailing outcomes after thoracic re-RT. In the present study, 89.6% of patients were retreated with conventionally fractionated 3D-CRT/IMRT. Due to patients with two initial pathologic types (NSCLC and SCLC), with various clinical diagnosis at re-RT (including recurrent, metastatic, or a new primary lung tumor), and only 38.8% of patients with histological confirmation at re-RT, we did not assessed the effectiveness of re-RT.

The CTCAE 4.0 and South-West Oncology Group (SWOG) criteria are frequently used for RP grading in studies. The differences between CTCAE 4.0 and SWOG criteria are mainly in grade 1 and 2 RP. Grade 1 RP graded according to SWOG criteria would change to grade 2 RP if the CTCAE 4.0 criteria would have been applied. There is no obvious difference in grade 3, 4, or 5 RP between the two criteria. In the present study, RP was graded according to the CTCAE 4.0, and rates of grade ≥ 3 RP were 26.9%. Similar finding was also reported in several studies of re-RT with SBRT [6, 14, 15]. Liu et al. [15] analyzed 72 patients receiving re-irradiation with SBRT for recurrent disease and found that rates of severe RP were common (20.8%). Data from a retrospective study conducted at MD Anderson showed that 28% of re-RT patients developed grade 3 RP, and RP was the most common side effect [6]. De Bari et al. analyzed 12 studies on thoracic re-irradiation with SABR after a previous course of high-dose radiotherapy and found that the major toxicity is RP, occurring in around 20% of patients [21]. Ruysscher et al. summarised the available literature-based evidence, and stated that the incidence of severe lung toxic effects did not differ greatly in patients retreated with conventional 3D-CRT compared with patients retreated with SBRT [22]. We also compared the risk of grade ≥ 3 RP between re-RT with conventional fractionation and with SBRT, while no significant difference was observed.

As we know, there is only one retrospective study conducted by Liu et al. [15] that has investigated DVH variables related to RP from 3 sets of plans (initial RT, re-RT, and composite plans). In their study, a wide range of DVH parameters (including V10-V40 and MLD of 3 sets of plans) were assessed, but only a V20 ≥ 30% in the composite plan was significantly correlated with the incidence of grade 3–5 RP in multivariate analysis. In the present study, DIR was performed to create a composite plan, and the correlation between DVH variables from 3 sets of plans (including V5, V20, V50, and MLD) and the risk of grade ≥ 3 RP were evaluated. Different from Liu et al’s results, we found that initial-MLD and composite-V5 were significantly associated with grade ≥ 3 RP in multivariate analysis. One possible explanation for the difference was difference in re-RT technique used for re-RT. All of patients were retreated with SBRT in Liu et al’s study, while most of patients (88.1%) were retreated with conventional 3D-CRT/IMRT in the present study. The unique fractionation schemes and the dose distribution of SBRT might make it different from conventional RT for dose-volume predictors of severe RP. Secondly, DIR was not used in Liu et al’s study when generating the composite plans, which might result in less accurate assessment of the cumulative radiation doses to lung. DIR is an image processing technique, with the potential to account for anatomic changes, including changes in body weight, and early or late normal tissue radiation responses [16, 17]. Our results suggested that not only re-RT but also initial-RT and composite plans should be considered as important options for evaluating retreatment with thoracic RT.

One previous study has shown that patients retreated with RT for in-field relapses experienced lower rates of RP than did those outside the initial radiation field (0% vs. 28%, *P* = 0.03) [6]. For reason of the difference, the authors hypothesized that previously irradiated areas have already undergone fibrosis and are less susceptible to radiation-induced inflammation. In the present study, relatively lower rates of grade ≥ 3 RP for in-field failures compared with out-of-field failures were observed (18.2% vs. 35.3%), but with no significant statistical difference (*P* = 0.114). Interestingly, we also found that in-field failures were more frequently observed in patients with higher overlap-V5/re-V5 (0.8–1) and out-of-field failures in patients with lower overlap-V5/re-V5 (0–0.8); and linear-by-linear association testing suggested a significant correlation between location of recurrent tumors (in-field/out-of-field) and overlap-V5/re-V5 (0.8–1/0–0.8) (*P* < 0.001). Moreover, we found that overlap-V5/re-V5 was an independent predictor of grade ≥ 3 RP in multivariate analysis. However, it was not that the larger overlap-V5/re-V5 was, the lower incidence of grade ≥ 3 RP became. Rates of grade ≥ 3 RP for patients with the largest overlap-V5/re-V5 of 0.8–1 were similar to patients with the smallest overlap-V5/re-V5 of 0–0.4 (18.3% vs. 14.3%, *P* = 1.000), but were significantly lower when compared with patients with the medium overlap-V5/re-V5 of 0.4–0.8 (18.3% vs. 50%, *P* = 0.014). These findings suggested that the overlap of volume of lung exposed to low radiation doses between initial RT plans and re-RT plans was associated with the incidence of severe RP, and should be paid more attention when evaluating a plan of re- RT. Moreover, the findings also suggested an indirect association between location of recurrent tumors (in-field/out-of-field) and RP risk via the variable of overlap-V5/re-V5. Not all out-of-field relapses were associated with higher risk of severe RP compared with in-field relapses, and the correlation seemed to be determined by size of overlap-V5/re-V5.

ECOG PS score of 2–3, an FEV1 ≤ 65% were reported to be associated with higher RP in Liu et al.’s study [15]. In our study, none of patients were with ECOG PS score of 3, and there was no significant difference in RP risk between ECOG PS score of 0–1 and 2; the correlation between FEV1 and RP risk was not investigated because of lack of related data. Other potential risk factors of RP such as chemotherapy, the interval between initial and re-RT, location of re-RT (central/peripheral) were also assessed in this study, however, none of them were predictive for severe RP.

There were several limitations associated with this study. First, this is a retrospective, single-institution study, and biases could have been introduced which would affect the conclusions. The multivariate analysis can aid in decreasing these biases but not necessarily negate them entirely. Second, the sample size of this study was relatively small. This makes statistical analysis less reliable and raises the possibility that other important risk factors could be missed. Finally, a few patients who received very low doses of initial RT or re-RT were also included, which might lead to less accurate on the assessment of RP rates after re-RT.

## Conclusions

The risk of grade ≥ 3 RP could be predicted by initial-MLD, composite-V5, and overlap-V5/re-V5. Out-of-field failures was associated with higher risk of severe RP compared with in-field failures in some cases, however, the correlation seemed to be determined by size of overlap-V5/re-V5.

## Abbreviations

3D-CRT: Three-dimensional conformal radiotherapy
4D-CT: 4-dimensional computed tomography
CT: Computed tomography
DIR: Deformable image registration
DVH: Dose-volume histogram
GTV: Gross tumor volume
iGTV: Internal gross tumor volume
IMRT: Intensity modulated radiotherapy
ITV: Internal target volume
MLD: Mean lung dose
PTV: Planning target volume
Re-RT: Reirradiation
RP: Radiation pneumonitis
RT: Radiotherapy
SBRT: Stereotactic body radiation therapy
Vx: Percent volume of lung exposed to at least x Gy
